# Understanding the Difference Between Self-Feedback and Peer Feedback: A Comparative Study of Their Effects on Undergraduate Students' Writing Improvement

**DOI:** 10.3389/fpsyg.2021.739962

**Published:** 2021-09-13

**Authors:** Qi Lu, Xinhua Zhu, Choo Mui Cheong

**Affiliations:** ^1^College of Education, Zhejiang University, Hangzhou, China; ^2^Department of Chinese and Bilingual Studies, The Hong Kong Polytechnic University, Kowloon, Hong Kong, SAR China; ^3^Faculty of Education, University of Hong Kong, Pokfulam, Hong Kong, SAR China

**Keywords:** self-feedback, peer feedback, feedback amount, feedback types, feedback implementation

## Abstract

Due to the growing popularity of Assessment for Learning in higher education, self- feedback and peer feedback are often highlighted for their role in improving writing performance. In order to provide appropriate support for students' effective implementation of the feedback, teachers must comprehend the differential characteristics of self- and peer feedback. However, empirical research comparing the two remains scarce, particularly when they are used in combination. In this study, 116 Hong Kong undergraduate students participated in an abstract writing task and engaged in self- and peer feedback processes. The amount, types, and implementation of self- and peer feedback and their effects on writing improvement were analyzed and compared. Hierarchical regression analyses indicated that about 25% of the variance in the students' writing improvement was collectively accounted for by the two feedback processes. One form of feedback contributed about 15% of the variance while the other form explained 10%. Feedback types and the amount of implemented feedback were found to be positive predictors of writing improvement, whereas the overall feedback amount negatively affected the improvement. Moreover, the implementation of peer feedback was found to have a greater effect on the improvement than those of self-feedback. Several pedagogical implications of these findings are addressed.

## Introduction

As alternative means of assessment gain increasing popularity in higher education, self- and peer feedback (also known as formative self- and peer assessment) are often highlighted for their roles in improving authentic assessment performance (Boud and Soler, 2016; Adachi et al., 2018) and supporting learning (Sadler, 1989; Topping, 2003). Carless characterized feedback as the “dialogic processes whereby learners make sense of information from various sources and use it to enhance their work or learning strategies” (Carless, 2016, p.1). This definition emphasizes the connection between feedback process (i.e., generating feedback) and its outcome (i.e., achieving educational gains), which capitalizes on the benefits of feedback as a means to improve learning. In the context of writing, self- feedback involves the activities during which learners reflect upon their own writing, and revise in subsequent drafts, while peer feedback covers practices of providing and receiving comments and suggestions from peers and make improvement at the receival end. Given that recent studies scrutinize feedback in terms of the amount, type and implementation (Patchan et al., 2016; Wu and Schunn, 2020), self- and peer feedback are operationalized by the feedback amount, types and the amount of implemented feedback.

Researchers have argued that self- and peer feedback play different roles in improving writing and should therefore be integrated into writing classrooms alongside each other to maximize their effects (Suzuki, 2008; Lam, 2013; Hung et al., 2016; Ndoye, 2017). Although some empirical studies have compared the impacts of self- and peer feedback (Diab, 2011), what the differences between the feedback processes are and how diverse the features that facilitate writing outcomes are remain unclear. The lack of a comprehensive understanding of the effectiveness of self- and peer feedback for writing outcomes hinders adequate support for students to engage in effective feedback processes (Noroozi et al., 2016). In addition, comparative studies have been conducted in quasi-experimental settings (Birjandi and Siyyari, 2010), with self- and peer feedback processes assigned to different experimental groups. The findings of such research may not explain students' choices when both feedback modes are available. In this study, we aim to fill this research gap by combining the use of self- and peer feedback in real classroom contexts. We empirically explore how self- and peer feedback differ, both during the feedback process and in their effects on writing outcomes.

## Literature Review

### Comparing the Effects of Self- and Peer Feedback on Writing Performance

Several studies have compared the effects of self-feedback and peer feedback on essay revisions. Some studies have found more revisions in response to peer feedback (Berger, 1990; Johnson, 2012), whereas others have discovered more revisions in response to self-feedback (Paulus, 1999; Suzuki, 2008; Diab, 2011). A few studies investigating the effects of these feedback modes on writing performance have yielded mixed findings. Some have indicated that peer feedback is more effective than self-feedback (Birjandi and Siyyari, 2010; Fathi and Khodabakhsh, 2019), whereas others have concluded the opposite (Ozogul et al., 2008; Nielsen, 2019). Notably, such studies have been outcome-oriented, meaning that they did not compare the process of self-feedback to that of peer feedback. Such a comparison would shed more light on writing performance.

Two studies compared the amount and type of feedback generated in self- and peer feedback processes, as well as their impact on writing performance (Ozogul et al., 2008; Ozogul and Sullivan, 2009). The researchers concluded that more informative comments were generated in peer feedback, which therefore enhanced writing performance more than self-feedback (Ozogul and Sullivan, 2009). Although these studies seemingly point toward that peer feedback provides more benefits than self-feedback, the studies compared the two feedback processes generated by two different groups of students. We therefore would not know what similar or different impact they may have on writing when both kinds of feedback are available. To the best of our knowledge, no studies have carried out a detailed comparison spanning from process to outcome in which the two kinds of feedback were used concurrently.

### Feedback Amount, Types, Implementation, and Writing Performance

Recent studies on feedback processes have used variables such as amount, type, and implementation of feedback, to examine their effects on writing (Patchan et al., 2016; Huisman et al., 2017; Wu and Schunn, 2020). The first two variables describe the generating process of the feedback, while the third is addressed as an intermediate node between the feedback process and feedback outcome. However, these variables have solely been examined in relation to one kind of feedback (e.g., self-, peer, or teacher feedback). We review studies regarding the variables' relationships to writing performance; the results can be found below.

Feedback “amount” refers to the number of feedback comments that students generate. Several studies have found a positive relationship between feedback amount and its subsequent implementation, indicating that students tend to implement and make more revisions when they receive more feedback (Tsui and Ng, 2000; Cho and MacArthur, 2010; Patchan et al., 2013; Wichmann et al., 2018). More feedback probably leads to a wider range of information tied to writing that students can acquire. Other studies have also drawn opposing conclusions. Patchan et al. (2016) demonstrated the negative impact of the amount of peer feedback on its implementation. Similar conclusions have been reached in the field of marketing education. Ackerman and Gross (2010) discovered that receiving a higher number of comments from teachers raised students' concerns about their performance on the writing assignment on marketing strategy. The students became doubtful of themselves, which led to less willingness to implement the feedback. Consequently, feedback became less effective due to the students' negative reaction to it (Carless, 2006). While the above studied on the effect of feedback amount on feedback implementation, research on whether the amount of feedback accounts for writing improvement is scarce. We found one such study conducted by Wu and Schunn (2020). The researchers revealed that amount of feedback has an indirect effect on improvement in writing. However, if the feedback are of low quality, the improvement can be minimal. These findings suggest that feedback amount, although is an indicator for feedback, may not be adequate as a standalone. Therefore, more indicators should be considered.

Feedback “type” refers to the classification of feedback based on its content and focus. The most common types are content-related, function-related, and presentation-related feedback (Narciss and Huth, 2004; Narciss, 2008; Shute, 2008). Content-related types of feedback usually provide feedback on prose issues (i.e., language use and coherence) and substance (i.e., issues with writing content; Inuzuka, 2005; Cho and Cho, 2011; Patchan et al., 2013; Ene and Upton, 2014). Function-related feedback types refer to the role that feedback serves concerning the text. This type of feedback has been categorized into analysis, evaluation, explanation, and revision (Van den Berg et al., 2006; Van der Pol et al., 2008; Huisman et al., 2017, 2018). Others have classified feedback as cognitive or affective; they have identified cognitive feedback categories of summarization, specificity, explanation, and scope and affective feedback categories of praise and mitigation language (Nelson and Schunn, 2009; Patchan and Schunn, 2015; Patchan et al., 2016). Finally, presentation-related feedback may contain information about timing (i.e., immediate vs. delayed), schedule (i.e., simultaneous vs. sequential), adaptivity (i.e., adaptive vs. non-adaptive), and form (i.e., single-medium vs. multi-medium).

While explaining and analyzing, the function-related feedback types usually provides the receiver of the feedback information regarding the writing review process (Flower et al., 1986), and is useful in enhancing students' learning, we thus adopt this categorization of feedback in the study. We further select summary, praise, problems, and solutions as our feedback types for two reasons. First, they are the most common specific feedback types in the function-related categorization discussed above. Second, each type has been found to have a significant influence on writing. Summary and praise have been found to be positively associated with feedback implementation (Bienstock et al., 2007; Nelson and Schunn, 2009); problems and solutions have been found to have inconsistent results regarding their relationships with writing across different studies (Tsui and Ng, 2000; Tseng and Tsai, 2007; Bitchener, 2012; Huisman et al., 2017). These inconsistent results may be attributed to students' varying writing proficiencies. While the above studies have often examined each specific type's impact on writing (Nelson and Schunn, 2009; Patchan et al., 2016), our current study aims to illuminate the overall effect of the varied feedback types.

The implementation of feedback is seen as the articulation connecting the feedback process and outcome. It describes the action that incorporates the feedback in the revision (Wu and Schunn, 2020). As reviewed above, most studies have focused on its association with the feedback process (Nelson and Schunn, 2009), rather than on its relationship with the outcome. It seems that the positive relationship between feedback implementation and writing improvement has been naturally admitted. In fact, as it refers to the change students introduce into the text (Guasch et al., 2019), it does not necessarily draw forth the improvement of the writing quality. The text change can either be good or bad. Its effect on writing would be influenced by various factors, including the feedback source (Suzuki, 2008; Diab, 2011), feedback quality (Tseng and Tsai, 2007; Nelson and Schunn, 2009), students' abilities regarding the feedback literacy (Jonsson, 2013), and their cognition of feedback implementation (Winstone et al., 2017). We further scrutinize the implementation of feedback from different sources, since the respective effect of self- and peer feedback implementation on students' writing improvement remain unclear when the two feedback modes are used concurrently.

In sum, there are three main research gaps in the literature. First, little research comparing self- and peer feedback that relates the feedback process to its outcomes, especially when the two kinds of feedback are used in integration, has been conducted. It is vital for teachers to have a comprehensive understanding of the differences between the self- and peer feedback processes and their respective effects on writing if they are to support students in conducting effective feedback. Second, few studies have explored the effect of feedback on writing when self- and peer feedback are used concurrently. Most studies have explored the impact of only one mode of feedback (Patchan et al., 2016). Finally, very little research has inquired into the relationship between the two feedback modes by incorporating feedback amounts, types and implementation, with writing improvement. We innovatively use these diverse variables, and we investigate how they are illustrated in self- and peer feedback and how they may affect students' writing improvement.

## The Study

The study was part of a larger project that explored the characteristics of multiple means of assessment (e.g., self- and peer feedback) and their effects on improving undergraduate students' academic writing. In light of the gaps in knowledge arising from prior studies, the present study aims to explore the difference in amount, types, and implementation between self- and received peer feedback and to examine the differential effects of the two feedback modes on students' writing improvement. Accordingly, the following research questions are addressed:

RQ1: What are the differences between self- and peer feedback in terms of feedback's amount, types, and implementation?
RQ2: To what extent do feedback's amount, types, and implementation predict students' writing improvement?

## Methods

### Participants

The participants recruited were Bachelor of Arts (Hons) students studying in language communication sciences in Hong Kong. The students were enrolled in an academic writing course, which was designed to enrich their knowledge of research methods and enhance their academic writing skills. Since the Assessment for Learning was introduced as an initiative for curriculum reform in Hong Kong in the early 2000s (Curriculum Development Institute, 2004), it has been gradually well-entrenched as a crucial pedagogy used in higher education. Self- and peer assessment thereby have increasingly been advocated in the writing classrooms within the context of higher education. The participants were therefore familiar with self- and peer assessment, as these practices are common in education in Hong Kong. There were 116 students (Male = 26, Female = 90; Mean age = 21.97, SD = 1.04) taking the course. All of the students signed a consent form indicating their voluntary participation.

### Materials

#### Abstract writing task

Abstract writing was one of the essential learning tasks in academic writing. The students were invited to read an academic article titled, “The challenges in teaching non-Chinese speaking students in Hong Kong Chinese language classrooms” (Kwan, 2014), which was published in the journal Newsletter of Chinese Language (now called Current Research in Chinese Linguistics). The article was selected for the following reasons: it was a research paper but without an abstract, and the topic discussed in the paper was relevant to the academic writing course.

#### Abstract Writing Scoring Rubrics

The rubric used to evaluate the students' abstract writing performance was adapted from Tankó (2017). It comprised five dimensions: research purpose, research method, research findings, implications, and language convention. This rubric was chosen to be the measure of the abstract writing for two reasons. First, it is a commonly used structure of abstracts that fulfill the requirements of the academic journals in the field of social sciences with the IMRAD (Introduction-Methods-Results-Discussion) structure (Lorés, 2004; Alexandrov and Hennerici, 2007). To make it more explicit for undergraduates, we replaced “introduction” with “research purpose” and “discussion” with “implications.” Second, as indicated by Weil (1970) and Tankó (2017), a good structured abstract is expected to be communicative and informative, with increased readability and searchability. We therefore added the criteria on language convention into the rubric to examine whether students are competent to express coherently and concisely in their writing. This is the key to make the abstract readable and searchable. Each dimension was further divided into five fine-grained levels; each level ranged across two marks. The highest level was 7–8, illustrating excellent performance (e.g., research purpose is described clearly, concisely, and correctly, with a clear understanding of the aim of the paper), and the lowest level was 0, representing null or inappropriate/unacceptable performance (e.g., research purpose is missing or inappropriately described). Appendix A provides the rubric details.

#### Self- and Peer Feedback Forms

To facilitate the students' reviews of their own and their peers' writing, two identical forms were created. One form was for self-feedback and the other for peer feedback. There were two fields in each form: the first field required the students to review how they or their peers wrote according to the abstract writing scoring rubric, and the second required the students to state their suggestions for improvement. Each field was sub-divided into the same five dimensions as the abstract writing scoring rubric.

### Procedure

The four-step procedure took place in two lessons over 2 weeks.

1. Write an abstract: During the first lesson, the course teacher spent 50 min teaching the students how to write an academic abstract and introducing the scoring rubric for abstract writing. The students were asked to use this rubric when they reviewed their own or their peers' writing. Next, the students were given 80 min to read the aforementioned article, write their first draft of the abstract, and submit their work to the electronic course learning platform.
2. Review their own writing: After submission, 20 min were given for students to complete a self-feedback form on the platform. The form asked them to comment on the five dimensions of writing based on the scoring rubric of an abstract displayed on the platform.
3. Review a peer's writing: A week later, at the start of the second lesson, the students were randomly divided into groups of two through the course learning platform. They were given 30 min to read their partner's abstract and provide feedback. They referred to the same five-dimension scoring rubric used in Step (2).
4. Revise their own text based on their self-feedback and feedback received from their peers: Finally, the students were given another 50 min to read the self-feedback and the peer feedback they received and to revise their first drafts of the abstract. Final drafts were submitted to the platform when the students completed their revisions.

### Marking and Coding

#### Abstract Writing Quality

The second and third authors of this study, each with over 10 years of experience teaching academic writing, rated the first and final drafts according to the scoring rubric. To establish the accuracy and consistency of the scoring, the two raters met to discuss the task and rubric guidelines in detail. Both raters then scored a random subset of 60 first drafts and 60 final drafts. A mean score was assigned if there was only a small difference (i.e., <2 marks) between the two scores. However, when the discrepancies between the scores were greater than two, a third rater, a faculty member who taught the same course, was invited to rate the abstract. The third rater's score was matched with the closest score assigned by the original raters, and an average score was taken. The inter-rater reliability for double-rated data was calculated using the intraclass correlation coefficient. The calculation resulted in 0.88 for the first draft and 0.82 for the second draft, demonstrating a good estimate of reliability. The rest of the abstracts were rated independently. The improvement in writing quality was then taken to be the score of the final draft subtracted by that of the first draft.

#### Feedback Coding

The first author and a trained research assistant with a Master's degree conducted the feedback coding. All the authors met with the two coders to discuss the feedback coding scheme and conduct a trial with 10 randomly selected sets of self- and peer feedback before the actual coding. The results were discussed in detail to ensure that the two coders agreed on how to use the feedback coding scheme consistently. After the trial, another 60 sets of feedback were randomly selected and coded. The inter-rater reliability for the data was calculated using Cohen's kappa. All disagreements were discussed until a consensus was reached, to reduce the coding noise. The remaining self- and peer feedback comments were coded by the primary coder. The coding consisted of feedback amount, feedback types, and amount of feedback implemented. Details are provided below.

##### Feedback Amount

**Figure 1:**
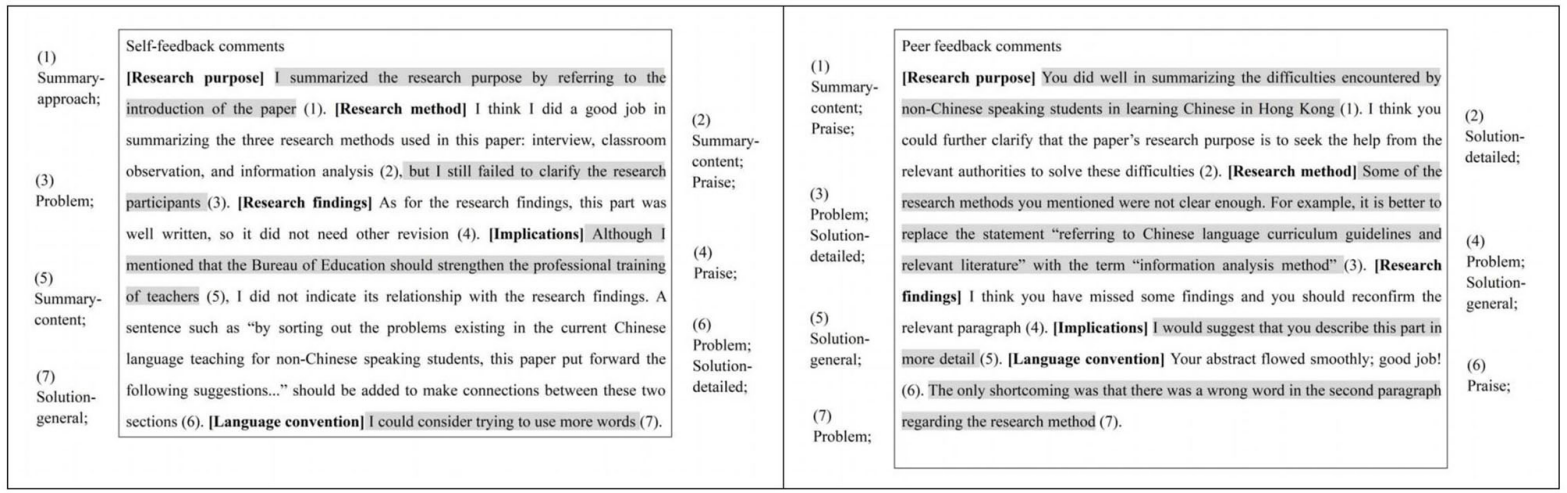
Examples of feedback amount and feedback types.

To determine the amount of feedback generated in self- and peer feedback, the feedback comments were first segmented into idea units. An idea unit is a comment that centers on a particular issue with the students' writing (Patchan et al., 2016; Wu and Schunn, 2020). It may range from a few words to several sentences that describe a complete idea. In general, one dimension from the rubric could include one or more idea units. For instance, one set of self-feedback comments (presented in Figure 1 below) was segmented into seven units within the five dimensions of the rubric. It was further subdivided into two units within the research method dimension. One unit described what the author had written and another unit stated the problems in the text.

A total of 1,479 idea units in self-feedback and 1,404 idea units in peer feedback were produced during feedback segmentation. The inter-rater reliability estimate was the kappa value of 0.85 and 0.78 for the self- and peer feedback comments, respectively. The amount of self-feedback and peer feedback per student was also calculated.

##### Feedback Types

A categorization of feedback types comprising summary, praise, problem, and solution was used, adapted from Nelson and Schunn (2009) and Patchan et al. (2016). Each feedback comment was first coded into these four types. The summary segment was further coded according to whether it was a content summary or an approach summary, and the solution segment was classified as a detailed solution or a general solution. Appendix B provides more details about each feedback type.

Each student could therefore produce six types of feedback, although they did not always do so. After the feedback was divided into units, each unit was analyzed for its feedback types. One unit could contain one or more types of feedback. As an example, see the peer feedback comments shown in Figure 1. The third unit (attributed to the research method dimension) described an idea concerning a term-usage issue. This idea was characterized as a problem and a detailed solution. The respective number of feedback types in self- and peer feedback per student was subsequently calculated for analysis.

##### Amount of Feedback Implemented

Comments segmented as problems and/or solutions were regarded as implementable. There are situations where students detect a problem without providing a solution. In this case, some of them may attempt revisions based on the problem, although they are not sure whether the revision is effective. Matsumura et al. (2002) found that students tended to revise their writing when they identified problems. There were 739 and 509 comment units in self- and peer feedback, respectively, that were found to be implementable (excluding vague comments). Subsequently, the two coders analyzed the actual implementation of feedback within all implementable comments. Appendix C provides more details. Two steps were taken to determine the students' actual implementation of feedback. First, the first and final drafts were compared using the “Compare Documents” function in Microsoft Word to identify the revisions made in the final drafts. Changes in format were not considered. Second, the source of the revision was examined by locating the relevant comments in either the self- or peer feedback. Note that a comment in the self- and/or peer feedback was recognized as “Implemented” if a revision was found related to it; otherwise, it will be labeled as “Not Implemented.” Furthermore, <3% of the implementable comments were coded as vague implementation. These comments were usually presented as general statements without distinct directions for improvement; it could not be determined whether they were implemented (e.g., “Reread the text several times”). These comments were therefore eliminated from the analysis. A total of 437 comment units in self-feedback and 332 comment units in peer feedback were found to have been implemented. The inter-rater reliability of coding each self- and peer feedback comment implemented was the kappa value of 0.75 and 0.66 (percentage agreement was 70%), respectively. Afterwards, the data were processed and analyzed at the level of the amount of feedback implemented per student.

### Data Analysis

The data collected from abstract writing and its relevant coding results were input into SPSS 25.0 for four stages of statistical analysis. Descriptive statistics, including the minimum, maximum, mean, standard deviation, skewness, and kurtosis, were carried out to examine the data's central tendencies, variation, and distributional characteristics. Subsequently, a paired-samples *t*-test was performed to compare the difference between self- and peer feedback. Pearson product-moment correlation analysis was then executed to measure the relationships among amount, types, and implementation of self- and peer feedback and students' writing improvement. Lastly, a hierarchical linear regression analysis based on the result of the correlation analysis was conducted to explore the potential effects of these feedback-related variables on writing improvement. To examine the respective contributions of self- and peer feedback, we entered each feedback in different orders and formed two models to be analyzed. The prerequisite assumptions required by the regression analysis, including linear relationships, outliers, normal distribution, homoscedasticity, and multicollinearity, were examined before regression. The results showed that there was no independent variable (i.e., feedback amount, types, and amount of feedback implemented in self- and peer feedback) with the value of variance inflation factor (VIF) <2. This figure was below the cutoff threshold of 10, indicating these variables would not confound the regression results (Hair et al., 2014).

## Results

### Descriptive Statistics

Table 1 shows the descriptive statistics results. The mean self- and peer feedback values were 12.75 and 12.10 for feedback amount, 4.66 and 4.59 for feedback types, and 3.77 and 2.86 for amount of feedback implemented, respectively. The mean scores on the final draft (26.07) were higher relative to the first draft (22.31) [*t*(115) = 14.98, *p* < 0.001], indicating that almost all of the students improved in their writing after the self- and peer feedback processes. The minimum writing improvement was 0, as six students had not improved their writing after the feedback process. In addition, the absolute values of skewness and kurtosis ranged from 0.01 to 2.51 and from 0.04 to 8.73, respectively, showing a reasonably normal data distribution (Kline, 2016).

**Table 1:** Descriptive statistics for measured variables (*N* = 116).

	**Min**.	**Max**.	**M**	**SD**	**Skewness**	**Kurtosis**
Self-feedback (SF)
Feedback amount_SF	7	21	12.75	3.01	0.73	0.24
Feedback types_SF	2	6	4.66	0.98	−0.76	0.41
Summary-content_SF	0	5	3.61	1.42	−0.85	−0.10
Summary-approach_SF	0	5	1.48	1.77	0.82	−0.82
Praise_SF	0	5	1.86	1.45	0.52	−0.64
Problem_SF	0	5	2.62	1.56	−0.11	−1.11
Solution-detailed_SF	0	5	1.01	1.30	1.50	1.80
Solution-general_SF	0	5	3.45	1.51	−0.96	−0.04
Amount of SF implemented	0	9	3.77	2.24	0.01	−0.77
Peer feedback (PF)
Feedback amount_PF	7	21	12.10	2.91	0.71	0.21
Feedback types_PF	2	6	4.59	0.94	−0.16	−0.56
Summary-content_PF	2	5	4.31	0.83	−0.92	−0.11
Summary-approach_PF	0	4	0.36	0.67	2.51	8.73
Praise_PF	0	5	3.60	1.11	−0.63	0.17
Problem_PF	0	5	1.85	1.43	0.51	−0.54
Solution-detailed_PF	0	5	1.56	1.31	0.56	−0.18
Solution-general_PF	0	5	1.82	1.47	0.37	−0.99
Amount of PF implemented	0	10	2.86	2.18	1.06	1.20
Writing performance
Writing performance of first draft	15	30	22.31	3.58	0.08	−0.63
Writing performance of final draft	19	36	26.07	3.40	0.12	0.24
Writing improvement	0	13	3.76	2.71	1.01	1.24

### Difference Between Self- and Peer Feedback

In response to research question 1 on the difference between self- and peer feedback, the results of the pairwise comparison between self- and peer feedback are shown in Table 2. Significant differences between the two modes of feedback were found in the feedback amount, feedback types, and amount of feedback implemented. For this study, effect size was computed by Cohen's *d* as it is an indicator of practical and academic significance (Alias et al., 2015). The absolute values of Cohen's *d* ranged from 0.20 to 0.97, indicating a small to large practical importance of the finding (Cohen, 1988; Kirk, 1996), besides the total number of feedback types [*t*(115) = 0.69, *p* = 0.49, Cohen's *d* = 0.07]. The feedback amount was higher for self-feedback and summary-approach, problem, and general solution feedback types were more common. Summary-content, praise, and detailed solution feedback types were more common in peer feedback. A greater amount of self-feedback was found to be implemented [*t*(115) = 4.10, *p* < 0.001, Cohen's *d* = 0.38] in contrast with peer feedback implementation. The results revealed the differences between self- and peer feedback processes in terms of all the variables, i.e., feedback amount, feedback types and implemented feedback, indicating that students have different performance as self- and peer reviewers.

**Table 2:** Paired *t*-test for self- and peer feedback (*N* = 116).

**Self-feedback (I)**	**Peer-feedback (J)**	**Mean difference *(I-J)***	***SE***	***t***	***p***	**Cohen's *d***
Feedback amount_SF	Feedback amount_PF	0.65	3.29	2.12	^*^	0.20
Feedback types_SF	Feedback types_PF	0.08	1.22	0.69	0.49	0.07
Summary-content_SF	Summary-content_PF	−0.70	1.56	−4.83	^***^	0.45
Summary-approach_SF	Summary-approach_PF	1.12	1.79	6.76	^***^	0.63
Praise_SF	Praise_PF	−1.74	1.80	−10.40	^***^	0.97
Problem_SF	Problem_PF	0.77	1.62	5.11	^***^	0.48
Solution-detailed_SF	Solution-detailed_PF	−0.55	1.60	−3.71	^***^	0.34
Solution-general_SF	Solution-general_PF	1.63	1.87	9.40	^***^	0.87
Amount of SF implemented	Amount of PF implemented	0.91	2.38	4.10	^***^	0.38

*** *p < 0.001,*

* *p < 0.05*.

### Relationship Between Self- and Peer Feedback and Writing Improvement

The results of the correlational analysis of the seven variables are shown in Table 3. As expected, feedback types and amount of feedback implemented were both positively correlated with writing improvement. However, the amount of self- and peer feedback was found to have a weakly negative but non-significant correlation with writing improvement (*r* = −0.11 for SF, *r* = −0.02 for PF). In addition, all of the absolute correlation coefficients between the seven variables were not strong, ranging from 0.02 to 0.50, implying the inclusion of these variables in the regression model was possible. We then use this model to examine the respective effects of self- and peer feedback processes on students' writing improvement.

**Table 3:** Bivariate correlations of self- and peer feedback and writing improvement (*N* = 116).

	**1**	**2**	**3**	**4**	**5**	**6**	**7**
1 Feedback amount_SF	1						
2 Feedback types_SF	0.44^**^	1					
3 Amount of SF implemented	0.04	0.14	1				
4 Feedback amount_PF	0.38^**^	0.21^*^	0.24^*^	1			
5 Feedback types_SF	0.29^**^	0.20^*^	0.20^*^	0.37^**^	1		
6 Amount of PF implemented	0.21^*^	0.15	0.42^**^	0.50^**^	0.26^**^	1	
7 Writing improvement	−0.11	0.16	0.31^**^	−0.02	0.18^*^	0.31^**^	1

* *p < 0.05;*

** *p < 0.01*.

Regarding research question 2 on how self- and peer feedback predict students' writing improvement, the results of the hierarchical regression analysis are reported in Table 4. Regardless of the order of entering each kind of feedback in the regression model, one mode of feedback predicted around 15% of the variance in writing improvement in Step 1^1^, and the other mode in Step 2^2^ accounted for a further 10% of the variance. As a result, a total of about 25% of the variance in overall writing improvement (*p* < 0.001) in the two models was jointly explained by self- and peer feedback. This implied when used simultaneously, self- and peer feedback's amount, types, and amount of feedback implemented will account for 25% of writing improvement. On one hand, by comparing the standardized regression coefficients (Beta) of each variable in self- and peer feedback in the full model presented in Step 2, the discrepancies between the Beta values of the amounts (−0.24, −0.24) and types (0.21, 0.18) of self- and peer feedback were both < 0.04, indicating that self-feedback amount and peer feedback amount had similar effects on the improvement outcome, as did feedback types. On the other hand, the comparison between self- and peer feedback implementation showed significant differences. The implementation of peer feedback had a greater effect on writing improvement than that of self-feedback, with a Beta value of 0.32 for the former and 0.18 for the latter. Lastly, all variables in self- and peer feedback significantly predicted writing improvement. Feedback amount was a negative contributor to the improvement outcome variance, while feedback types and the amount of feedback implemented were positive contributors.

**Table 4:** Hierarchical regression analysis predicting writing improvement with self- and peer feedback (*N* = 116).

	**Step 1 (β)**	**Step 2 (β)**
**Model 1**
Feedback amount_SF	−0.22^*^	−0.24^*^
Feedback types_SF	0.22^*^	0.21^*^
Amount of SF implemented	0.29^**^	0.18^+^
Feedback amount_PF		−0.24^*^
Feedback types_PF		0.18^+^
Amount of PF implemented		0.32^**^
*R* ^2^	0.152^***^	0.252^***^
Δ*R*^2^		0.101^**^
**Model 2**
Feedback amount_PF	−0.28^**^	−0.24^*^
Feedback types_PF	0.18^+^	0.18^+^
Amount of PF implemented	0.40^***^	0.32^**^
Feedback amount_SF		−0.24^*^
Feedback types_SF		0.21^*^
Amount of SF implemented		0.18^+^
*R* ^2^	0.159^***^	0.252^***^
Δ*R*^2^		0.093^**^

+ *p < 0.06;*

* *p < 0.05;*

** *p < 0.01;*

*** *p < 0.001*.

Note that there is an inconsistency between the results of correlation analysis (i.e., the amount of self- and peer feedback was found to have a weakly negative but non-significant correlation with writing improvement) and regression analysis (i.e., the amount of self- and peer feedback significantly predicted writing improvement). It may be owed to the different functions of these two analyses. The correlation analysis only measures the relationship between two variables regardless of the possible impact of other variables on the outcome, whereas the regression measures the relationship between two variables with consideration of the influence of other variables. As indicated by Costa (2017), the coefficient of each variable in the regression model is known as a partial correlation. This difference may explain in the case that occurred in this study, there is no significant correlation is found between feedback amount and writing improvement in the correlation analysis, but a significant prediction from the feedback amount on writing improvement in the regression analysis is detected.

## Discussion

In this study, we compare the differences between self- and peer feedback processes and explore the effects of the two modes of feedback on students' writing improvement. Our major findings are discussed below.

### The Differences Between Self-Feedback and Peer Feedback

With respect to research question 1, more self-feedback than peer feedback was produced. This result is inconsistent with the findings of Ozogul et al. (2008) and Ozogul and Sullivan (2009). A plausible explanation for this contradiction may be due to the different requirements for the feedback to be provided in different studies. As introduced in the procedures made by Ozogul and Sullivan (2009, p. 399): “in Week 3, students are trained on conducting lesson plan evaluations and on providing constructive feedback.” This type of feedback mainly asked students to write comments about errors in the text and/or corresponding solutions. This requirement of the feedback content may have resulted in the students writing less self-feedback as it is harder to identify problems in their own writing than in the work of others. However, in our study, the students were allowed to comment on specific aspects of the abstract. Their comments could include but were not limited to error detection and/or solution selection. This freedom thus increased the likelihood that they would generate more comments.

As for feedback types, the following observations deserve discussion. First, more summary-content comments were found in peer feedback. Summary-approach comments appeared more in self-feedback. These results may be attributed to the nature of these two feedback types. According to Kritikos et al. (2011), self-reviewing students tend to evaluate whether they have conveyed their intended message instead of what they achieved in their text. The likelihood of generating summary-approach comments that record students' reflection on their writing process is therefore higher than that of producing summary-content comments outlining their actual written content in self-feedback. However, the same does not hold for peer feedback. The actual written content, rather than the writing process behind it, is the foundation for peer feedback. Student peer reviewers judge how well an essay is written by evaluating whether the content meets the requirements stipulated in the assessment rubric. Consequently, we discovered more summary-content comments in peer feedback. The difference in the summary sub-types between self- and peer feedback implied that students may experience different cognitive processes when acting as self-reviewers and peer reviewers, respectively. Future research could further explore the differences between the cognitive processes of self- and peer feedback and the causes of these differences.

Second, more praise was found in peer feedback compared to self-feedback. There could have been two reasons for this. First, stringency with oneself and leniency toward others are often observed in collectivist cultures (Atwater et al., 2009). This means that the students might have been more inclined to compliment others in peer feedback. Second, a belief in the principle of reciprocity might have driven the students to give more praise in their peer feedback to receive reciprocal appreciation from their peers, as Koponen and Nousiainen had found in their study “that individuals appreciate each other in collaborative or task-oriented groups in which mutual trust or benefit is assumed to regulate social interactions” (Koponen and Nousiainen, 2016, p. 17).

Third, more general solutions and problems were observed in self-feedback, but detailed solutions were produced more in peer feedback. This finding suggests that the students could provide detailed solutions for others but might have been limited in their ability to do so in self-feedback. According to Flower et al. (1986), errors in text are more likely to be ignored by self-reviewers because they will automatically mentally correct them. This finding implies that it is not necessary for students to generate detailed solutions for problems, as they would have already been considered solved. Consequently, less detailed, but more general solutions appear in self-feedback. It is worth noting that this result found in Flower et al. (1986) study did not conflict with our finding that more amount of feedback was found in self-feedback. Feedback amount was computed by units that center on different issues, which means that less ability in generating one particular type of feedback (i.e., detailed solutions) would not reduce the total amount of feedback. In fact, as students are familiar with their own writing, they generated comments on other aspects of writing other than detailed solutions for problems. Whereas, when reviewing others' work; students provide detailed solutions to particular problems (Mendonça and Johnson, 1994). This asymmetry might have led to our finding that more detailed solutions appeared in peer feedback. Furthermore, as the students reflected upon their writing before receiving the feedback from their peers, when the detailed solutions they received compliment their own feedback that contains general solutions and problems, there may be a higher tendency that they will follow up on the feedback.

### The Effect of Self- and Peer Feedback on Writing Improvement

Regarding research question 2, the amounts of both self- and peer feedback had a negative influence on writing improvement. According to Patchan et al. (2016), excessive feedback probably burdens students' working memory (Sweller, 1994; Van Merrienboer and Sweller, 2005) as they need to judge the feedback's usefulness for their writing. Moreover, students may find it difficult to understand feedback with many units due to time constraints; they may simply ignore such feedback.

The types of both self- and peer feedback were found to affect writing improvement positively. This is opposite to the results of the study on reading comprehension by Kulhavy et al. (1985). They pointed out that students benefited more from fewer feedback types (e.g., only providing correct answers) when trying to improve their reading performance and efficiency. This contradiction might be due to the type of task that the students were faced with (i.e., multiple-choice questions). Fewer types of feedback could mean that the students had more time to correct other questions and achieve their reading goals. However, in the field of writing, A piece of feedback containing varied types is well-articulated and presents a more complete logic for the writer. Well-articulated feedback includes an overall reviewing process that crystallizes task defining, problem detecting, problem diagnosing, and revision strategies selection (Flower et al., 1986; Hayes et al., 1987), along with positive motivational beliefs (Nicol and Macfarlane-Dick, 2006). When a piece of feedback has all of these elements, it is more convincing and encouraging. It therefore increases students' willingness to implement it. Conversely, when students are given insufficient types of feedback, they may lack the necessary information to understand the gap between their current and desired performance or the relationship between problems and solutions.

The required specifics in feedback were logic-interlocking, including all of the elements mentioned above. This was one of the study's major contributions. To scaffold students' learning, effective feedback should be a purposeful and connected reaction to a range of issues in their writing rather than a collection of aimless and unorganized comments (Martin, 2010; Mahboob, 2015). In other words, feedback should clarify for students what its purpose is (i.e., to improve the quality of writing) and how it conveys its message concerning the purpose in a coherent manner. Mahboob (2015) once identified two ways to achieve the coherence in feedback: “degree of explicitness (i.e., whether feedback provided explicit correction of errors)” (p. 408) and “amount of rationale (i.e., whether feedback provided explanation about errors)” (p. 409). The study plotted a Cartesian plane with four quadrants representing four feedback types (i.e., hand holding, carrying, bridging, and base jumping). Our results enrich the required feedback types and further clarify that achieving such coherence requires providing a range of feedback types that form a complete logic.

**Figure 2:**
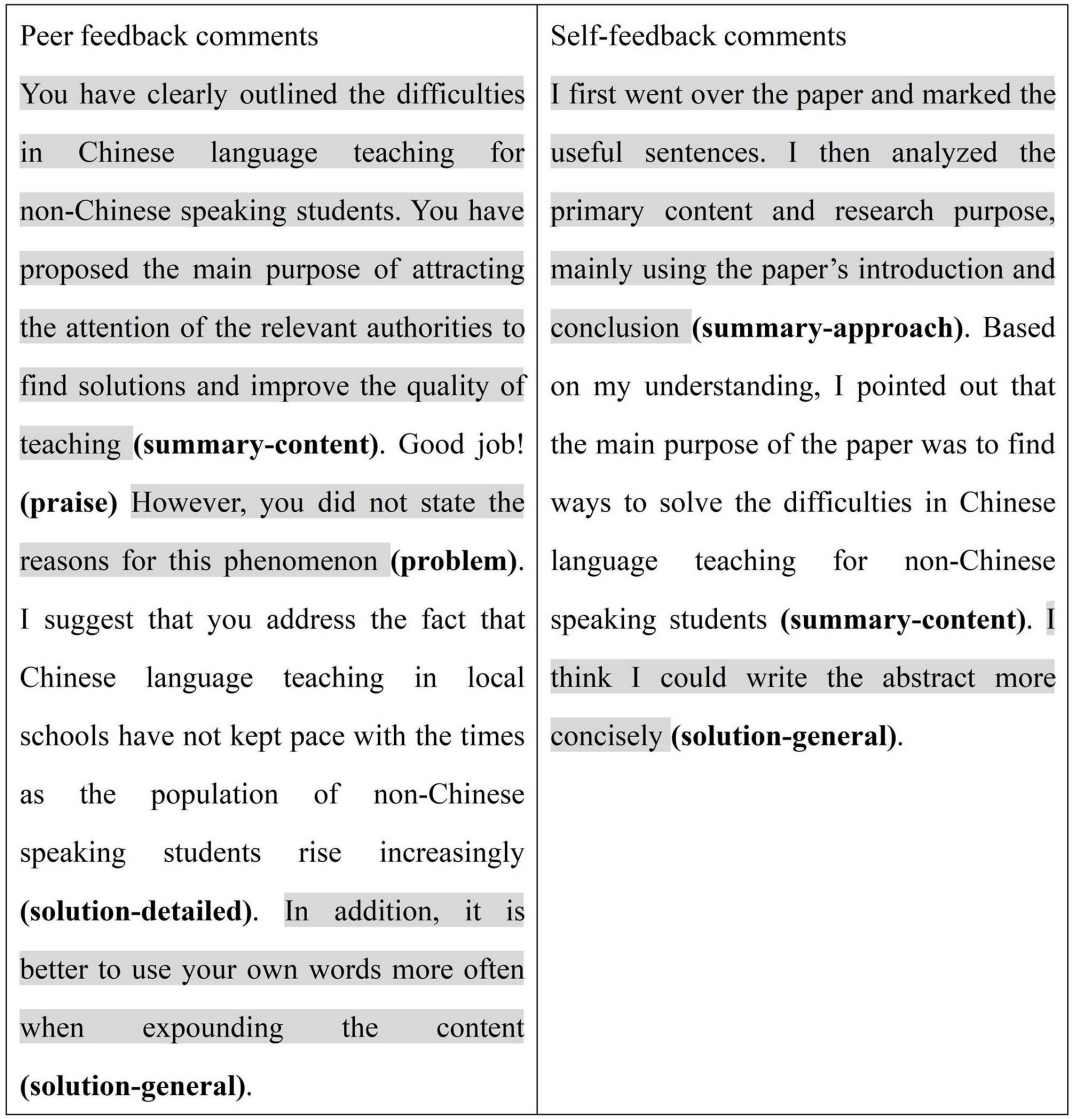
Examples of comments containing different feedback types.

Take the example on the left of Figure 2. The user of this feedback would be able to follow the thoughts of the feedback provider, with understanding what the current performance was, what they were doing well, why and/or where their problems lay. Therefore, he or she would like to implement the corresponding solutions to resolve the problem, making a better work. An alternative solution (i.e., solution-general) was also provided to show the student another way to improve the quality of the writing. However, the feedback on the right provided mostly summaries; it simply ended with a general solution without addressing any specific problems. As expected, the user of these pieces of feedback mainly implemented the feedback on the left instead of the feedback on the right. The user thus improved the quality of writing.

The amount of feedback implemented from the two modes of feedback was proven to have a significant effect on writing improvement. Furthermore, a relatively greater contribution from peer feedback implementation to the overall improvement was found. This greater contribution may be attributed to the quality differences between implemented self- and peer feedback. As mentioned in the literature review, the effect of feedback implementation on writing would be affected by the feedback quality (Tseng and Tsai, 2007; Nelson and Schunn, 2009). Nelson and Schunn (2009) argued that comments that provide specific information about a problem or a solution reduce ambiguity and improve understanding of the identified problem. These comments thus elicit writing improvements. In our case, more detailed solutions were observed in the peer feedback. They provide explicit suggestions for correcting errors and thus improving writing quality. Hence, although the amount of feedback implemented was significantly higher in self-feedback than that in peer feedback, the regression analysis result indicated that peer feedback implementation is more effective in improving writing performance than self-feedback.

## Limitations, Conclusion, and Implications

Some limitations of our study should be noted. First, we did not specify what specific feedback types attributed the writing improvement, future research could investigate on the effect of a various array of feedback types. Second, although the design of arranging the same group of students to participate in both self- and peer feedback helped us compare the differences between the two feedback modes, we could not exclude the potential influence of self-feedback practice on the following peer feedback practice. As Zong et al. (2020) indicated, the prior experience of feedback would impact on the quality of the next feedback practice. Future research could extract this linear effect and used counter-balanced measures to nuance it when self- and peer feedback are used concurrently. Finally, the sample size did not support further analysis of other factors, like gender differences.

We contribute a more integrative understanding of differing feedback processes and their relationships to writing improvement through a real classroom situation where self- and peer feedback were conducted concurrently. More importantly, we fill a gap in the literature by associating feedback amount, types, and implementation in different feedback modes with writing improvements. Our major findings include that self- and peer feedback differed in amount, specific types, and implementation. Higher amounts of feedback were found in self-feedback, and more feedback was implemented. Self-feedback also contained more approach-summary, problem, and general solution types. More content-summary, praise, and detailed solution types were found in peer feedback. Furthermore, the number of feedback types and amount of implemented feedback positively predicted writing improvement, but the amount of feedback negatively predicted improvement. Finally, peer feedback implementation was comparatively more effective in improving students' quality of writing.

We draw three major pedagogical implications for writing classrooms from these findings. First, simply having more fragmented feedback may not benefit students. Feedback containing more complete logic is instead helpful for accomplishing students' goals on improving the writing. Quality rather than quantity should be emphasized in the feedback process. Explicit instruction on how to provide highly logical feedback that contains the overall reviewing process (Flower et al., 1986; Hayes et al., 1987) and motivational beliefs (Nicol and Macfarlane-Dick, 2006) is needed to develop students' ability to write effective comments. Second, given the observation of a positive influence of feedback implementation on writing improvement, enhancing students' feedback literacy is vital. The ability to read, interpret, and use given feedback affects the effectiveness of its implementation (Sutton, 2012). An exemplar analysis is one learning activity that would promote students' feedback literacy. Teachers could organize dialogues on exemplars chosen from students' feedback to illustrate the essay's assessment standards. Such dialogues would scaffold students' academic judgement of the quality of work (Carless and Chan, 2017; Carless and Boud, 2018). Lastly, a combination of self- and peer feedback is recommended. Teachers could instruct students to conduct self-feedback first and then ask them to concurrently consider their self-feedback while undergoing a round of peer feedback to optimize their performance.

## Data Availability Statement

The original contributions presented in the study are included in the article/Supplementary Files, further inquiries can be directed to the corresponding author.

## Ethics Statement

The studies involving human participants were reviewed and approved by Department of Chinese and Bilingual Studies, The Hong Kong Polytechnic University. The participants provided their written informed consent to participate in this study.

## Author Contributions

XZ and CC designed the methodologies of the research project. QL and XZ collected and analyzed the data collaboratively. QL drafted the initial manuscript (including the figures and tables). CC and XZ later elaborated and revised the manuscript while maintaining close communication with QL. All authors contributed to the article and approved the submitted version.

## Conflict of Interest

The authors declare that the research was conducted in the absence of any commercial or financial relationships that could be construed as a potential conflict of interest.

## Publisher's Note

All claims expressed in this article are solely those of the authors and do not necessarily represent those of their affiliated organizations, or those of the publisher, the editors and the reviewers. Any product that may be evaluated in this article, or claim that may be made by its manufacturer, is not guaranteed or endorsed by the publisher.

## Footnotes

^1^Step 1 indicated the entry of one kind of feedback (i.e., self- or peer feedback) for regression analysis, including this feedback' amount, types, and amount of feedback implemented.

^2^Step 2 indicated the entry of the other kind of feedback for regression analysis. For example, if self-feedback was entered as Step 1, Step 2 will enter peer feedback into the model; and vice versa. Step 2 also showed the full model that included self- and peer feedback.

## References

[B1] Ackerman D. S. Gross B. L. (2010). Instructor feedback: how much do students really want? J. Mark. Educ. 32, 172–181. 10.1177/0273475309360159

[B2] Adachi C. Tai J. H. M. Dawson P. (2018). Academics' perceptions of the benefits and challenges of self and peer assessment in higher education. Assess. Eval. High. Educ. 43, 294–306. 10.1080/02602938.2017.1339775

[B3] Alexandrov A. V. Hennerici M. G. (2007). Writing good abstracts. Cerebrovas. Dis. 23, 256–259. 10.1159/000098324 17199082

[B4] Alias M. Masek A. Salleh H. H. M. (2015). Self, peer and teacher assessments in problem based learning: are they in agreements? Proc. Soc. Behav. Sci. 204, 309–317. 10.1016/j.sbspro.2015.08.157

[B5] Atwater L. Wang M. Smither J. W. Fleenor J. W. (2009). Are cultural characteristics associated with the relationship between self and others' ratings of leadership? J. Appl. Psychol. 94:876. 10.1037/a0014561 19594231

[B6] Berger V. (1990). The effects of peer and self-feedback. CATESOL J. 3, 21–35.

[B7] Bienstock J. L. Katz N. T. Cox S. M. Hueppchen N. Erickson S. Puscheck E. E. (2007). To the point: medical education reviews—providing feedback. Am. J. Obstet. Gynecol. 196, 508–513. 10.1016/j.ajog.2006.08.021 17547874

[B8] Birjandi P. Siyyari M. (2010). Self-assessment and peer-assessment: a comparative study of their effect on writing performance and rating accuracy. Iran. J. Appl. Linguist. 13, 23–45.

[B9] Bitchener J. (2012). Written corrective feedback for L2 development: current knowledge and future research. TESOL Q. 46, 855–860. 10.1002/tesq.62

[B10] Boud D. Soler R. (2016). Sustainable assessment revisited. Assess. Eval. High. Educ. 41, 400–413. 10.1080/02602938.2015.1018133

[B11] Carless D. (2006). Differing perceptions in the feedback process. Stud. High. Educ. 31, 219–233. 10.1080/03075070600572132

[B12] Carless D. (2016). Feedback as dialogue, in Encyclopedia of Educational Philosophy and Theory, eds Peters M. A. (Cham: Springer), 1–6.

[B13] Carless D. Boud D. (2018). The development of student feedback literacy: enabling uptake of feedback. Assess. Eval. High. Educ. 43, 1315–1325. 10.1080/02602938.2018.1463354

[B14] Carless D. Chan K. K. H. (2017). Managing dialogic use of exemplars. Assess. Eval. High. Educ. 42, 930–941. 10.1080/02602938.2016.1211246

[B15] Cho K. MacArthur C. (2010). Student revision with peer and expert reviewing. Learn. Instruct. 20, 328–338. 10.1016/j.learninstruc.2009.08.006

[B16] Cho Y. H. Cho K. (2011). Peer reviewers learn from giving comments. Instruct. Sci. 39, 629–643. 10.1007/s11251-010-9146-1

[B17] Cohen J. (1988). Statistical Power Analysis for the Behavioral Sciences. Hillsdale, NJ: Erlbaum.

[B18] Costa V. (2017). Correlation and regression, in Fundamentals of Statistical Hydrology, ed Naghettini M. (Cham: Springer) p. 391–440. 10.1007/978-3-319-43561-9_9

[B19] Curriculum Development Institute (2004). Promoting Assessment for Learning in English Language Education. Hong Kong: Hong Kong Government Printer.

[B20] Diab N. M. (2011). Assessing the relationship between different types of student feedback and the quality of revised writing. Assess. Writ. 16, 274–292. 10.1016/j.asw.2011.08.001

[B21] Ene E. Upton T. A. (2014). Learner uptake of teacher electronic feedback in ESL composition. System 46, 80–95. 10.1016/j.system.2014.07.011

[B22] Fathi J. Khodabakhsh M. R. (2019). The role of self-assessment and peer-assessment in improving writing performance of Iranian EFL students. Int. J. Engl. Lang. Transl. Stud. 7, 1–10.

[B23] Flower L. Hayes J. R. Carey L. Schriver K. Stratman J. (1986). Detection, diagnosis, and the strategies of revision. Coll. Composit. Commun. 37, 16–55. 10.2307/357381

[B24] Guasch T. Espasa A. Martinez-Melo M. (2019). The art of questioning in online learning environments: the potentialities of feedback in writing. Assess. Eval. High. Educ. 44, 111–123. 10.1080/02602938.2018.1479373

[B25] Hair J. F. Black W. C. Babin B. J. Anderson R. E. (2014). Multivariate Data Analysis, 7th Edn. Upper Saddle River, NJ: Prentice Hall.

[B26] Hayes J. R. Flower L. Schriver K. A. Stratman J. F. Carey L. (1987). Cognitive processes in revision, in Advances in Applied Psycholinguistics, Vol. 2, ed Rosenberg S. (Cambridge: Cambridge University Press), 176–240.

[B27] Huisman B. Saab N. Van Driel J. Van den Broek P. (2017). Peer feedback on college students' writing: exploring the relation between students' ability match, feedback quality and essay performance. High. Educ. Res. Dev. 36, 1433–1447. 10.1080/07294360.2017.1325854

[B28] Huisman B. Saab N. Van Driel J. Van Den Broek P. (2018). Peer feedback on academic writing: undergraduate students' peer feedback role, peer feedback perceptions and essay performance. Assess. Eval. High. Educ. 43, 955–968. 10.1080/02602938.2018.1424318

[B29] Hung Y. J. Samuelson B. L. Chen S. C. (2016). Relationships between peer-and self-assessment and teacher assessment of young EFL learners' oral presentations, in Assessing Young Learners of English: Global and Local Perspectives, ed Nikolov M. (Cham: Springer), 317–338.

[B30] Inuzuka M. (2005). Learning how to write through encouraging metacognitive monitoring: the effect of evaluating essays written by others, in Paper Presented at the Annual Conference of the Cognitive Science Society (Stresa).

[B31] Johnson K. G. (2012). Peer and self-review: a holistic examination of EFL learners' writing and review process (Ph.D. dissertation). University of Arizona, Tucson, AZ, United States.

[B32] Jonsson A. (2013). Facilitating productive use of feedback in higher education. Active Learn. High. Educ. 14, 63–76. 10.1177/1469787412467125

[B33] Kirk R. E. (1996). Practical significance: a concept whose time has come. Educ. Psychol. Meas. 56, 746–759. 10.1177/0013164496056005002

[B34] Kline R. B. (2016). Principles and Practice of Structural Equation Modeling, 4th Edn. New York, NY: Guilford Press.

[B35] Koponen I. T. Nousiainen M. (2016). Formation of reciprocal appreciation patterns in small groups: an agent-based model. Complex Adapt. Syst. Model. 4:24. 10.1186/s40294-016-0035-6

[B36] Kritikos V. S. Woulfe J. Sukkar M. B. Saini B. (2011). Intergroup peer assessment in problem-based learning tutorials for undergraduate pharmacy students. Am. J. Pharm. Educ. 75:73. 10.5688/ajpe75473 21769149 PMC3138352

[B37] Kulhavy R. W. White M. T. Topp B. W. Chan A. L. Adams J. (1985). Feedback complexity and corrective efficiency. Contemp. Educ. Psychol. 10, 285–291. 10.1016/0361-476X(85)90025-6

[B38] Kwan C. Y. (2014). The challenges in teaching Non-Chinese speaking students in Hong Kong Chinese language classrooms [in Chinese]. Newslett. Chin. Lang. 93, 39–57.

[B39] Lam R. (2013). The relationship between assessment types and text revision. ELT J. 67, 446–458. 10.1093/elt/cct034

[B40] Lorés R. (2004). On RA abstracts: from rhetorical structure to thematic organisation. Engl. Spec. Purposes 23, 280–302. 10.1016/j.esp.2003.06.001

[B41] Mahboob A. (2015). Understanding and providing “cohesive” and “coherent” feedback on writing. Writ. Pedagog. 7, 401–422. 10.1558/wap.v7i2-3.26461

[B42] Martin J. R. (2010). Language, register, and genre, in Applied Linguistics, A Reader: Systemic Functional Linguistics, Critical Discourse Analysis, and Ethnography, eds Coin C. Lillis T. M. Halloran K.O' (New York, NY: Routledge), 12–32.

[B43] Matsumura L. C. Patthey-Chavez G. G. Valdés R. Garnier H. (2002). Teacher feedback, writing assignment quality, and third-grade students' revision in lower-and higher-achieving urban schools. Elem. Sch. J. 103, 3–25. 10.1086/499713

[B44] Mendonça C. O. Johnson K. E. (1994). Peer review negotiations: revision activities in ESL writing instruction. TESOL Q. 28, 745–769. 10.2307/3587558

[B45] Narciss S. (2008). Feedback strategies for interactive learning tasks, in Handbook of Research on Educational Communications and Technology, 3rd Edn., eds Spector J. M. Merril M. D. van Merriënboer J. J. G. Driscoll M. P. (Mahwah, NJ: Lawrence Erlbaum), 125–144.

[B46] Narciss S. Huth K. (2004). How to design informative tutoring feedback for multimedia learning, in Instructional Design for Multimedia Learning, eds Niegemann H. M. Leutner D. Brunken R. (Munster, NY: Waxmann), 181–195.

[B47] Ndoye A. (2017). Peer/self-assessment and student learning. Int. J. Teach. Learn. High. Educ. 29, 255–269.

[B48] Nelson M. M. Schunn C. D. (2009). The nature of feedback: how different types of peer feedback affect writing performance. Instruct. Sci. 37, 375–401. 10.1007/s11251-008-9053-x

[B49] Nicol D. J. Macfarlane-Dick D. (2006). Formative assessment and self-regulated learning: a model and seven principles of good feedback practice. Stud. High. Educ. 31, 199–218. 10.1080/03075070600572090

[B50] Nielsen K. (2019). Peer and self-assessment practices for writing across the curriculum: learner-differentiated effects on writing achievement. Educ. Rev. 1–22. 10.1080/00131911.2019.1695104

[B51] Noroozi O. Biemans H. Mulder M. (2016). Relations between scripted online peer feedback processes and quality of written argumentative essay. Internet High. Educ. 31, 20–31. 10.1016/j.iheduc.2016.05.002

[B52] Ozogul G. Olina Z. Sullivan H. (2008). Teacher, self and peer evaluation of lesson plans written by preservice teachers. Educ. Technol. Res. Dev. 56:181. 10.1007/s11423-006-9012-7

[B53] Ozogul G. Sullivan H. (2009). Student performance and attitudes under formative evaluation by teacher, self and peer evaluators. Educ. Technol. Res. Dev. 57, 393–410. 10.1007/s11423-007-9052-7

[B54] Patchan M. M. Hawk B. Stevens C. A. Schunn C. D. (2013). The effects of skill diversity on commenting and revisions. Instruct. Sci. 41, 381–405. 10.1007/s11251-012-9236-3

[B55] Patchan M. M. Schunn C. D. (2015). Understanding the benefits of providing peer feedback: how students respond to peers' texts of varying quality. Instruct. Sci. 43, 591–614. 10.1007/s11251-015-9353-x

[B56] Patchan M. M. Schunn C. D. Correnti R. J. (2016). The nature of feedback: how peer feedback features affect students' implementation rate and quality of revisions. J. Educ. Psychol. 108:1098. 10.1037/edu0000103

[B57] Paulus T. M. (1999). The effect of peer and teacher feedback on student writing. J. Second Lang. Writ. 8, 265–289. 10.1016/S1060-3743(99)80117-9

[B58] Sadler D. R. (1989). Formative assessment and the design of instructional systems. Instruct. Sci. 18, 119–144. 10.1007/BF00117714

[B59] Shute V. J. (2008). Focus on formative feedback. Rev. Educ. Res. 78, 153–189. 10.3102/0034654307313795

[B60] Sutton P. (2012). Conceptualizing feedback literacy: knowing, being, and acting. Innov. Educ. Teach. Int. 49, 31–40. 10.1080/14703297.2012.647781

[B61] Suzuki M. (2008). Japanese learners' self revisions and peer revisions of their written compositions in English. TESOL Q. 42, 209–233. 10.1002/j.1545-7249.2008.tb00116.x

[B62] Sweller J. (1994). Cognitive load theory, learning difficulty, and instructional design. Learn. Instruct. 4, 295–312. 10.1016/0959-4752(94)90003-525993279

[B63] Tankó G. (2017). Literary research article abstracts: an analysis of rhetorical moves and their linguistic realizations. J. Engl. Acad. Purposes 27, 42–55. 10.1016/j.jeap.2017.04.003

[B64] Topping K. (2003). Self and peer assessment in school and university: reliability, validity and utility, in Optimising New Modes of Assessment: In Search of Qualities and Standards, eds Segers M. Dochy F. Cascallar E. (Dordrecht: Kluwer Academic Publishers), 55–88.

[B65] Tseng S. C. Tsai C. C. (2007). On-line peer assessment and the role of the peer feedback: a study of high school computer course. Comput. Educ. 49, 1161–1174. 10.1016/j.compedu.2006.01.007

[B66] Tsui A. B. Ng M. (2000). Do secondary L2 writers benefit from peer comments? J. Second Lang. Writ. 9, 147–170. 10.1016/S1060-3743(00)00022-9

[B67] Van den Berg I. Admiraal W. Pilot A. (2006). Designing student peer assessment in higher education: analysis of written and oral peer feedback. Teach. High. Educ. 11, 135–147. 10.1080/13562510500527685

[B68] Van der Pol J. Van den Berg B. A. M. Admiraal W. F. Simons P. R. J. (2008). The nature, reception, and use of online peer feedback in higher education. Comput. Educ. 51, 1804–1817. 10.1016/j.compedu.2008.06.001

[B69] Van Merrienboer J. J. Sweller J. (2005). Cognitive load theory and complex learning: recent developments and future directions. Educ. Psychol. Rev. 17, 147–177. 10.1007/s10648-005-3951-0

[B70] Weil B. H. (1970). Standards for writing abstracts. J. Am. Soc. Inf. Sci. 21, 351–357. 10.1002/asi.4630210507

[B71] Wichmann A. Funk A. Rummel N. (2018). Leveraging the potential of peer feedback in an academic writing activity through sense-making support. Eur. J. Psychol. Educ. 33, 165–184. 10.1007/s10212-017-0348-7

[B72] Winstone N. E. Nash R. A. Rowntree J. Parker M. (2017). ‘It'd be useful, but I wouldn't use it': barriers to university students' feedback seeking and recipience. Stud. High. Educ. 42, 2026–2041. 10.1080/03075079.2015.1130032

[B73] Wu Y. Schunn C. D. (2020). The effects of providing and receiving peer feedback on writing performance and learning of secondary school students. Am. Educ. Res. J. 58, 492–526. 10.3102/0002831220945266

[B74] Zong Z. Schunn C. D. Wang Y. (2020). Learning to improve the quality peer feedback through experience with peer feedback. Assess. Eval. High. Educ. 46, 973–992. 10.1080/02602938.2020.1833179

