# Supplementary material for: Understanding the Difference Between Self-Feedback and Peer Feedback: A Comparative Study of Their Effects on Undergraduate Students' Writing Improvement
Source: Front Psychol. 2021 Sep 13;12:739962. doi: 10.3389/fpsyg.2021.739962 (PMC8476032; doi:10.3389/fpsyg.2021.739962)
Supplement: Supplementary file 1 [file Table_1.docx]

Supplementary Material

**Appendix A |** Abstract writing scoring rubric

| **Marks** | **Level descriptor** |
| --- | --- |
| **Research purpose** | |
| **7-8** | Research purpose is described clearly, concisely, and correctly, with a clear understanding of the paper used to write the abstract. |
| **5-6** | Research purpose is described clearly, with an appropriate understanding of the paper used to write the abstract. |
| **3-4** | Some of the information presented in the research purpose is not described clearly, with some understanding of the paper used to write the abstract. |
| **1-2** | Most of the information presented in the research purpose is not described clearly, with a deficient understanding of the paper used to write the abstract. |
| **0** | Research purpose is fully missing or described inappropriately. |
| **Research method** | |
| **7-8** | Important information presented in the research method (e.g., sample, process, data analysis, etc.) is described comprehensively, clearly, and concisely. |
| **5-6** | Most of the important information presented in the research method (e.g., sample, process, data analysis, etc.) is described clearly. |
| **3-4** | Some of the important information presented in the research method (e.g., sample, process, data analysis, etc.) is described clearly but some key points are missing. |
| **1-2** | Most of the important information presented in the research method (e.g., sample, process, data analysis, etc.) is not described clearly or it is missing. |
| **0** | Research method is fully missing or described inappropriately. |
| **Research findings** | |
| **7-8** | Research findings are described comprehensively, and their expression is clear and concise. |
| **5-6** | Research findings are described comprehensively, but their expression is not concise. |
| **3-4** | Some of the research findings are missing, and their expression is not clear and concise. |
| **1-2** | Most of the research findings are missing. |
| **0** | Research findings are fully missing or described inappropriately. |
| **Implications** | |
| **7-8** | Implications are concluded logically, and closely connected to the research findings. |
| **5-6** | Implications are concluded clearly, with some connection to the research findings. |
| **3-4** | Implications are concluded, but the expression is not clear or not connected to the research findings. |
| **1-2** | Most of the implications are missing or concluded incorrectly with no obvious connection to the research findings. |
| **0** | Implications are fully missing or concluded inappropriately. |
| **Language convention** | |
| **7-8** | Language convention is rigorous and objective, the expression is concise and accurate, and the vocabulary is rich and varied. |
| **5-6** | Language convention is objective, the expression is clear, and the vocabulary is appropriate, but some occasional mistakes are made. |
| **3-4** | Language convention is objective and the expression is correct, but there are some ill-formed sentences and inappropriate vocabularies. |
| **1-2** | Basic meaning can be understood, but there are many mistakes in the expression, negatively affecting the understanding of the abstract. |
| **0** | The expression is inappropriate, with many mistakes. |

**Appendix B |** Definition for feedback type

| **Category** | **Definition** |
| --- | --- |
| Summary  (k=0.70 for self-feedback, k=0.82 for peer feedback) | Statements of the current writing. |
| Content | Statements of content the author wrote. |
| Approach | Statements of approaches the author used in the writing process to express certain content. |
| Praise  (k=0.95 for self-feedback, k=0.88 for peer feedback) | Positive comments on good features in the writing. |
| Problem  (k=0.91 for self-feedback, k=0.92 for peer feedback) | Statements of something wrong with the writing. |
| Solution  (k=0.94 for self-feedback, k=0.84 for peer feedback) | Suggestions for resolving the problem or improving the quality of the writing. |
| Detailed | Explicit suggestions for resolving the problem or improving the quality of the writing. They were usually accompanied with a specific description of revisions (e.g., providing a revision example) and could be easily located in the writing. |
| Detailed | Implicit suggestions for resolving the problem or improving the quality of the writing. They were not necessarily related to a certain problem or could not be easily located in the writing. |

**Appendix C |** Coding scheme for feedback implementation

| **Category** | **Definition** | **Example** |
| --- | --- | --- |
| Implemented | Comments that were applied in the revision. | **SF:** I would add some statements regarding the participants, process, and data analysis of the research.  **First draft text:** This study obtained data through interviews, classroom observation, and information analysis.  **Final draft text:** This study obtained data through interviews with primary and secondary school teachers and non-Chinese speaking students, classroom observation conducted in primary, secondary, and special schools and special schools, and by combining the information analysis using the teaching materials and exercises provided by the teacher.  **PF:** I think you could add a summary of the full text in the ending and appeal to the relevant authorities to solve the problems addressed in the section of the research findings, echoing the research purpose.  **First draft text:**This paper therefore summarized the shortcomings of Chinese language teaching for non-Chinese speaking students.  **Final draft text:**By summarizing the current situation and shortcomings of Chinese language teaching for non-Chinese speaking students, this study was expected to draw the attention of the relevant authorities and solve the problems as soon as possible. |
| Not Implemented | Comments that were not applied in the revision | **SF:** I would like to add a statement such as “To improve the effectiveness of learning modern standard Chinese for non-Chinese speaking students.”  **Note:** The author did not actually implement the comment in the revision.  **PF:** I think you should summarize the four research purposes of the paper more concisely.  **Note:** The author did not make a revision other than adding an adjective (altering “hope to raise the attention of relevant administration departments to seek out the solutions” to “hope to raise the **strong** attention of relevant administration departments to seek out the solutions”), which was not thought to be matched with the comment. |
| Vague implementation | Comments that contained an implicit or vague description of a problem and/or solution, the implementation of which could not be determined. | **SF:** Maybe I could delete some unimportant information.  **PF:** Read the paper several times and try to understand its meaning. |
